# Community and Hospital Healthcare Use by Adults With and Without Intellectual and Developmental Disabilities in Ontario, Canada, During the First 2 Years of the COVID‐19 Pandemic

**DOI:** 10.1111/jir.13209

**Published:** 2025-01-28

**Authors:** A. Durbin, R. Balogh, E. Lin, L. Palma, L. Plumptre, Y. Lunsky

**Affiliations:** ^1^ Department of Psychiatry, Temerty Faculty of Medicine University of Toronto Toronto Ontario Canada; ^2^ Unity Health Toronto, MAP Centre for Urban Health Solutions Li Ka Shing Knowledge Institute, St. Michael's Hospital Toronto Ontario Canada; ^3^ ICES Toronto Ontario Canada; ^4^ Faculty of Health Sciences Ontario Tech University Unity Health Oshawa Ontario Canada; ^5^ Centre for Addiction and Mental Health (CAMH) Azrieli Adult Neurodevelopmental Centre Toronto Ontario Canada

**Keywords:** COVID‐19, healthcare, hospital, intellectual and developmental disabilities, physician visit, virtual care

## Abstract

**Background:**

This study describes the proportion of Ontario adults with and without intellectual and developmental disabilities (IDD) who used community‐ and hospital‐based healthcare in the first 2 years of the pandemic compared with the year pre–COVID‐19.

**Methods:**

Linked health administrative databases identified 87 341 adults with IDD and also adults without IDD living in Ontario, Canada. For each cohort, counts and proportions of adults who used different types of healthcare services were reported for the pre–COVID‐19 year (16 March 2019 to 14 March 2020) and the first two COVID‐19 years (15 March 2020 to 14 March 2021 and 15 March 2021 to 14 March 2022).

**Results:**

Compared with the year prior to COVID‐19, the proportion of adults with and without IDD who used health services was lower during the first COVID‐19 year, but the likelihood of all types of visits increased during the second year. The likelihood of using homecare and of being hospitalized nearly returned to pre‐pandemic levels. Virtual physician visits increased in each COVID‐19 year from 5.2% prior to the pandemic to 13.0% in year 1 and 58.7% in year 2. For all years, the proportion of adults who used each service type was higher for those with IDD than without IDD.

**Conclusions:**

For adults with and without IDD in Ontario, Canada, during the first two COVID‐19 years healthcare use decreased for all service types, except for virtual physician visits. In the second year, healthcare use increased but did not reach pre–COVID‐19 levels. In all years, adults with IDD were more likely to use services than other adults.

## 1 Introduction

Adults with intellectual and developmental disabilities (IDD) have more co‐occurring health issues (Cooper et al. 2015; Kinnear et al. 2018), take more medications (Stortz et al. 2014; McMahon, Hatton, and Bowring 2020), use more healthcare (Lunsky et al. 2019) and have higher rates of premature mortality than individuals without IDD (O'Leary, Cooper, and Hughes‐McCormack 2018). The COVID‐19 pandemic exacerbated pre‐existing health issues for this population. During the first year of the COVID‐19 pandemic, adults with cognitive disabilities including IDD were more likely than those without disabilities to report delayed medical care in medical settings and at home (Akobirshoev et al. 2022). Reduced healthcare contact and the rapid shift to virtual care were especially problematic for adults with IDD (Chadwick et al. 2022) because of their reliance on multiple healthcare providers (Kinnear et al. 2018) and the challenges they experienced adapting to pandemic restrictions (Keenan and Doody 2023; Taggart et al. 2022).

An earlier paper from our team (Durbin et al. 2022) compared health service use during the first COVID‐19 year to the preceding year in Ontario, Canada, for adults with and without IDD. It showed that service use decreased for all service types, except for virtual physician visits. For both years, adults with IDD remained more likely to use services than other adults, with the largest differences in use of mental health hospitalisations and mental health emergency department visits. This is consistent with results from the other settings that showed that during the pandemic, adults with IDD had reduced use of health and social care services during the first year of the pandemic (Flynn et al. 2022; Laufenberg et al. 2023).

Even though there were dramatic changes in availability and modality of delivery during the ongoing multiyear COVID‐19 pandemic (Kiran 2021), very little is known about healthcare use patterns of adults with IDD beyond the first year of the pandemic, either specifically or relative to the general population (Keenan and Doody 2023).

Two studies address this topic. One study that described service use beyond the first year of the pandemic for people with IDD focused on self‐reports of 694 adults with IDD and 447 carers of adults with severe or profound IDD from England, Northern Ireland, Scotland and Wales (Flynn et al. 2022; Hatton et al. 2024). During the first year and a half of the pandemic, many people with IDD who reported regularly accessing services pre‐pandemic reported not receiving them in any setting (e.g., in person, via the telephone or online) (Flynn et al. 2022). Although more in‐person visits occurred in 2022 than in 2020 or 2021, there continued to be problems accessing all types of healthcare. In 2022, people reported more cancellations of appointments and challenges getting their medications than previously. Nearly two thirds of participants (63%) reported not seeing the primary care provider they had seen previously. Subjective health ratings in 2022 were no better than ratings given at early stages of the pandemic, with some people reporting more difficulties (Hatton et al. 2024). Another longitudinal study (McCausland et al. 2021) of 682 Irish adults with IDD over 40 years of age also found reduced rates of healthcare. Between May and September 2021, most participants (58.2%) had not made any new healthcare appointments since the beginning of the pandemic; however, the participants did report an increase in phone/online consultations (22.2% to 48.0%). Neither of these studies used objective healthcare records from large datasets and instead relied on smaller samples of persons who reported on their (or their family member's) healthcare use. How the experience of these individuals varied from those without IDD was also not explored in either of these cohort studies.

### 1.1 Aim

This present study examined population level healthcare use changes for adults with and without IDD living in Ontario during each of the first 2 years of the COVID‐19 pandemic and the year prior, expanding on our prior study focused only on the first pandemic year. The first aim was to describe changes in healthcare use for adults with and for adults without IDD, during the first 2 years of the pandemic, compared with the preceding year. The second aim was to describe healthcare use for adults with IDD compared with adults with no IDD during the same time period.

## 2 Methods

### 2.1 Study Design and Setting

This is a population‐based retrospective cohort study of adults (18–105 years), who were alive and living in Ontario and eligible for provincial health insurance coverage as of 16 March 2019, with and without IDD identified. In Ontario, citizens and legal residents are eligible for the provincial health insurance plan, which provides universal coverage for basic and emergency healthcare services, including physician, ED and hospital care.

Healthcare use was examined from 16 March 2019 to 14 March 2020 (pre–COVID‐19 year), 15 March 2020 to 15 March 2021 (first COVID‐19 year) and 15 March 2021 to 15 March 2022 (second COVID‐19 year).

### 2.2 Data Sources and Linkage

Primary data sources were provincial administrative data sets (Appendix A) linked using unique, encoded identifiers and analysed at ICES. ICES is an independent, nonprofit research institute authorized under Ontario's privacy legislation to collect and analyse healthcare and demographic data, without consent, for health system evaluation and improvement. The use of data in this project was authorized under Section 45 of Ontario's Personal Health Information Protection Act, which does not require review by a research ethics board, and ICES is a prescribed entity according to this act. For more information on how ICES uses health administrative data for research and protects the privacy of individuals, please see https://www.ices.on.ca/Dataand‐Privacy/Privacy‐at‐ICES.

### 2.3 Study Cohort

There were 87 341 adults with IDD included in the study. Consistent with earlier research (Lin et al. 2013), if they had received a diagnosis of intellectual disability, foetal alcohol syndrome, autism and/or chromosomal and autosomal anomalies (e.g., Down syndrome) recorded during at 2+ physician visits or 1+ ED visit or 1 hospitalization since the date of their birth or database inception until 16 March 2019 and were alive and living in Ontario and eligible for Ontario Health Insurance Plan (OHIP) coverage as of 16 March 2019 (Appendix B). All other individuals were considered not to have IDD. Past research has shown that adults with IDD, compared with adults with no IDD, are much younger, more likely to be male and more likely to live in residences in the lowest income quintile (Durbin et al. 2022).

Outcomes were virtual and in‐office outpatient visits, home care, ED visits, mental health ED visits, hospitalisations and mental health hospitalisations (Table 1).

**TABLE 1:** Sociodemographic characteristics of adults as of 16 March 2019.

Characteristics		Adults with IDD (*n* = 87 341)	Adults without IDD (*n* = 12 070 363)
		*N* (%)	*n* (%)
Age group, in years	18–29	39 952 (45.7%)	2 252 489 (18.7%)
	30–39	14 715 (16.8%)	2 045 325 (16.9%)
	40–49	10 083 (11.5%)	2 013 337 (16.7%)
	50–59	10 627 (12.2%)	2 194 737 (18.2%)
	60–69	7287 (8.3%)	1 770 815 (14.7%)
	70–79	3212 (3.7%)	1 114 009 (9.2%)
	80–89	1164 (1.3%)	535 787 (4.4%)
	90+	301 (0.3%)	143 864 (1.2%)
Sex	Female	33 030 (37.8%)	6 173 050 (51.1%)
	Male	54 311 (62.2%)	5 897 313 (48.9%)
Rurality	Rural	10 315 (11.8%)	1 209 153 (10.0%)
	Urban	76 498 (87.6%)	10 827 853 (89.7%)
	Missing information	528 (0.6%)	33 357 (0.3%)
Residential income quintile	Missing information, *n* (%)	559 (0.6%)	37 623 (0.3%)
	1 (lowest)	24 681 (28.3%)	2 429 061 (20.1%)
	2	18 478 (21.2%)	2 411 667 (20.0%)
	3	15 763 (18.0%)	2 407 872 (19.9%)
	4	13 910 (15.9%)	2 381 548 (19.7%)
	5 (highest)	13 950 (16.0%)	2 402 592 (19.9%)

### 2.4 Analyses

For each of Ontario adults with and without IDD, we calculated the ratio comparing the proportion who used these services during each of the first two COVID‐19 years compared with the year prior to the pandemic. Ratios of 1.0 indicate similar levels of healthcare use across the two time periods. All statistical analyses were performed using SAS Version 9.4.

## 3 Results

Among adults with and without IDD visited ED, the proportion who were hospitalized and used home care was greater than the first year of the pandemic but lower than the year before it started, as shown in Table 2. Of adults with IDD, 60.2% had at least one in‐person physician visit during COVID's second year and the proportion who made at least one virtual physician visit was quite similar (58.7%). These patterns mirrored what occurred among adults without IDD.

**TABLE 2:** Non‐COVID outcomes for adults with DD 16 March 2019 to 14 March 2020 (pre–COVID‐19) and 15 March 2020 to 15 March 2022 (COVID‐19).

	Adults with IDD (*N* = 87 341)			Adults without IDD (*N* = 12 070 363)		
	Pre–COVID‐19	COVID‐19 Year 1	COVID‐19 Year 2	Pre–COVID‐19	COVID‐19 Year 1	COVID‐19 Year 2
In‐office physician visits, *n* (%)	71 060 (81.4%)	8970 (10.3%)	52 587 (60.2%)	9 178 276 (76.0%)	1 172 232 (9.7%)	7 167 594 (59.4%)
Mean (SD)	7.73 (9.12)	3.90 (6.01)	9.25 (13.46)	6.85 (7.10)	3.55 (5.56)	8.06 (9.22)
Median (Q1–Q3)	5 (2–10)	2 (1–4)	5 (3–11)	5 (2–9)	2 (1–4)	5 (3–10)
Virtual physician visit, *n* (%)	4497 (5.2%)	11 366 (13.0%)	51 279 (58.7%)	419 491 (3.5%)	1 505 231 (12.5%)	6 934 360 (57.5%)
Mean (SD)	3.27 (5.98)	2.56 (3.05)	10.78 (13.94)	2.78 (4.99)	2.51 (2.83)	9.11 (10.32)
Median (Q1–Q3)	1 (1–3)	2 (1–3)	7 (3–13)	1 (1–2)	2 (1–3)	6 (3–11)
Mental health ED visit, *n* (%)	6494 (7.4%)	2960 (3.4%)	5367 (6.1%)	141 311 (1.2%)	90 624 (0.8%)	123 071 (1.0%)
Any ED visit, *n* (%)	29 230 (33.5%)	10 685 (12.2%)	25 150 (28.8%)	2 624 576 (21.7%)	1 304 272 (10.8%)	2 349 123 (19.5%)
Any hospitalization, *n* (%)	9532 (10.9%)	5417 (6.2%)	8582 (9.8%)	720 051 (6.0%)	504 187 (4.2%)	666 594 (5.5%)
Mental health hospitalization, *n* (%)	3600 (4.1%)	1918 (2.2%)	3072 (3.5%)	43 805 (0.4%)	31 003 (0.26%)	41 617 (0.3%)
Homecare, *n* (%)	5150 (5.9%)	3237 (3.7%)	5024 (5.8%)	319 413 (2.7%)	227 138 (1.9%)	313 126 (2.6%)

**FIGURE 1:**
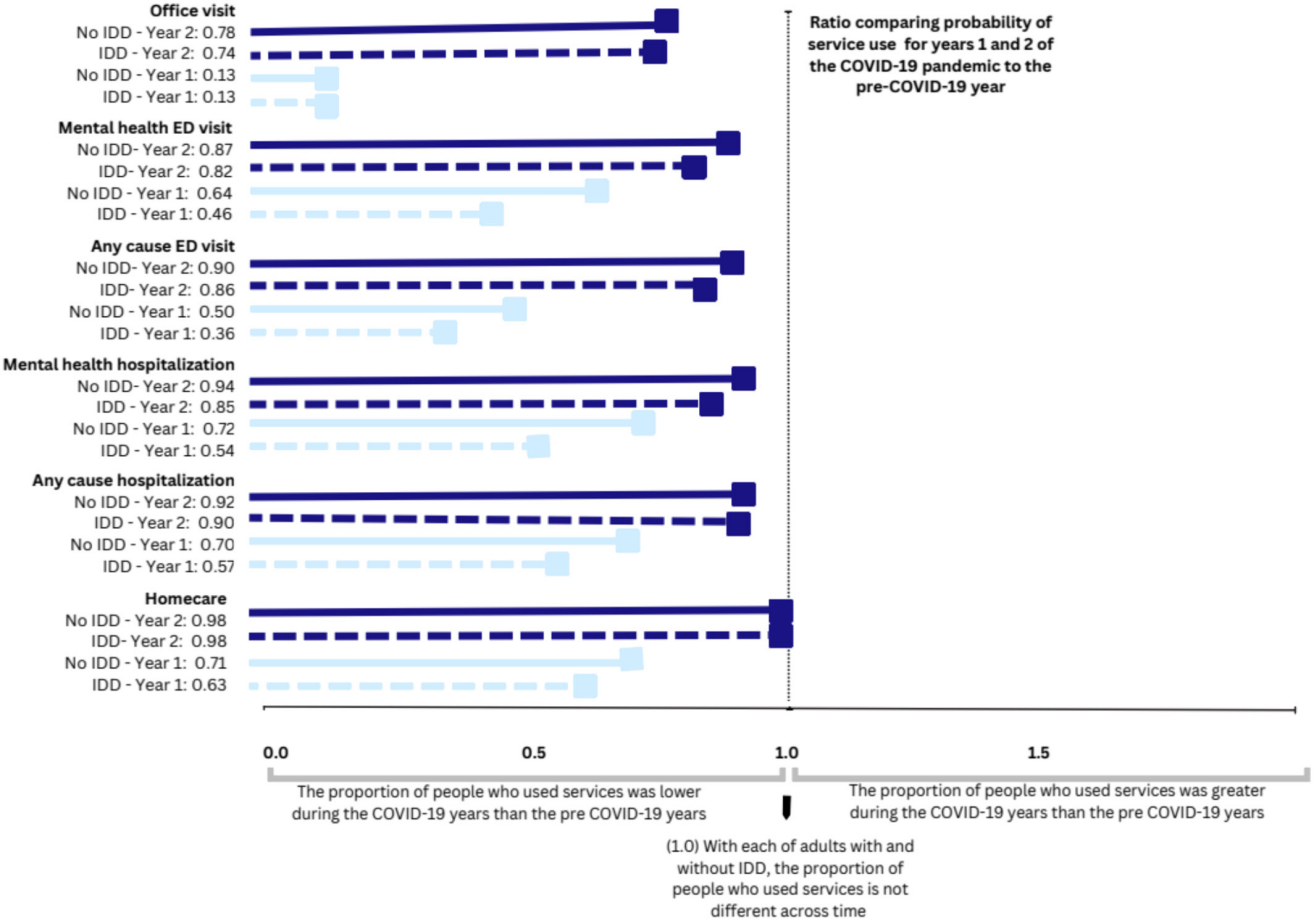
Ratios of the probability of having had any in‐person office physician visits and homecare, emergency department (ED) visit or hospitalization during the first and second COVID‐19 years, compared with the year that preceded COVID‐19, for adults with and without intellectual and developmental disabilities (IDD) in Ontario, Canada.

**FIGURE 2:**
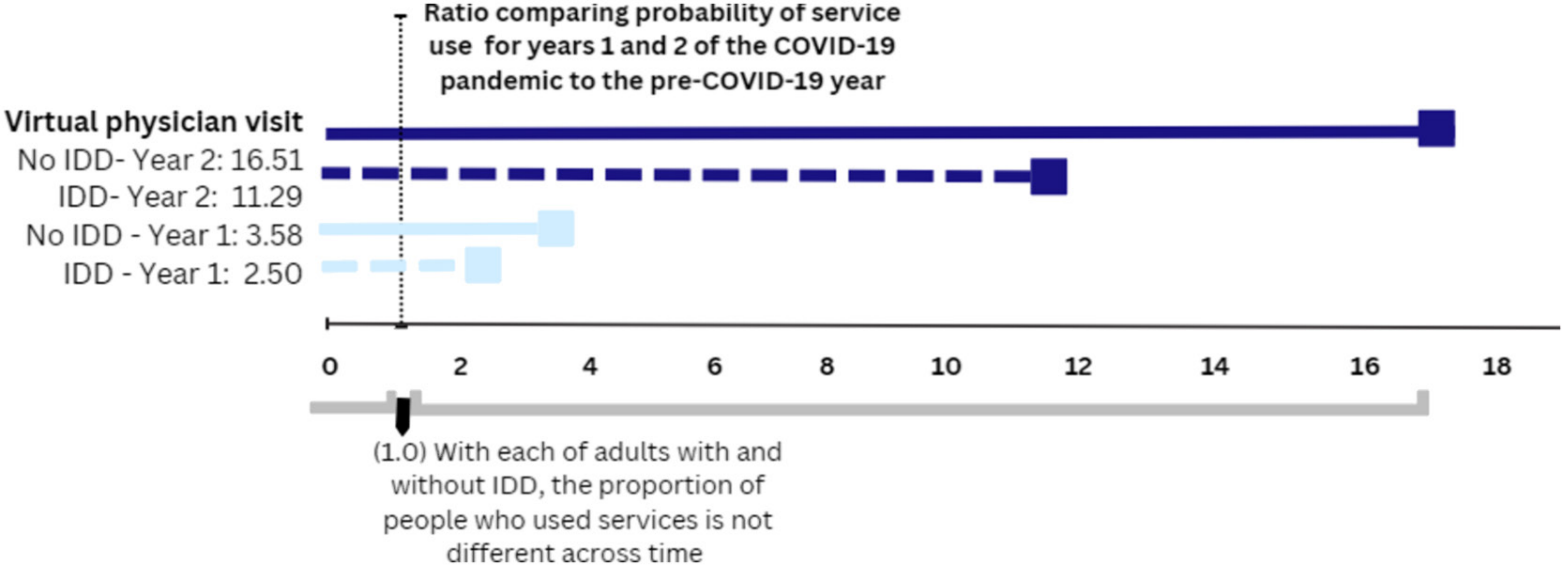
Ratios of the probability of having had any virtual physician visits during the first and second COVID‐19 years, compared with the year that preceded COVID‐19, for adults with and without intellectual and developmental disabilities (IDD) in Ontario, Canada.

For adults with and without IDD, ratios showed the probability of having made an in‐person office physician visits and homecare, ED visit or hospitalization (Figure 1) and virtual physician visits (Figure 2) during the first and second COVID‐19 years, compared with the year that preceded COVID‐19, in Ontario, Canada. With the exception of virtual physician visits, ratios are less than 1, which suggests lower probabilities of use in the years during the COVID‐19 pandemic compared with the preceding year. The only exception was virtual physician visits for which use increased during the pandemic.

## 4 Discussion

During the first year of COVID‐19, adults with IDD and without IDD were both less likely to use healthcare than the year prior to COVID‐19 across service types. During the second year, probability of service use increased for both cohorts, almost reaching pre–COVID‐19 levels for hospitalizations and homecare. The lone exception was that there was an increase in probability of making outpatient virtual visits during year 1 of the pandemic relative to the prior year, with even greater increases occurring in year 2. This aligns with the provincial government making these visits eligible for public insurance coverage in March 2020 (Glazier et al. 2021). Adults with IDD were more likely to use every type of healthcare than adults without IDD, although the extent of this difference varied by service type. The probability of making outpatient in‐office and virtual visits were very similar for adults with and without IDD in years 1 and 2 of the pandemic. However, ED visits for mental health reasons and hospitalisations for mental health reasons were much more common among adults with IDD during all 3 years studied.

The overall finding from our study is that healthcare use was returning to pre–COVID‐19 levels during the second year of the pandemic. Similar trends were observed for people with IDD and people with no IDD but with people with IDD remaining more likely to use healthcare. This population perspective, with the comparison group of all adults without IDD, offers a different picture than what was reported in smaller cohort studies based on self‐reported healthcare use. Research from Ireland (McCausland et al. 2021) and Scotland, England and Wales (Hatton et al. 2024) based on self and informant report noted continued healthcare gaps in the second year of the pandemic. Notably, neither of those studies nor our study reported increased uptake of healthcare resources among adults with IDD in year 2 relative to pre‐pandemic levels, which could have occurred if reduced access early in the pandemic led to even higher rates of need and subsequent use later. This could be considered a ‘good news’ story, but the current study does not capture how well unaddressed health needs were met once healthcare was more widely available. The long‐term impact of the pandemic on people should continue to be studied in case lags are more evident beyond the second year.

Despite in‐person care returning in the second year of the pandemic, rates of virtual care continued to increase for people with IDD and other individuals. This would suggest that for at least some individuals, virtual care became more accessible over time. Data from this study do not allow for comparisons in use across time for the same individual but provided estimates for the entire population. It is possible that some people may have only used one type of care or the other. Research on virtual healthcare in adults with IDD suggests that it can be an important accommodation for people who are fearful of becoming ill in public settings, as well as for people who have a hard time waiting at their appointments, or who get very anxious in healthcare settings (Lunsky et al. 2021; Selick et al. 2022). At the same time, both individuals with IDD, families and staff have spoken about how virtual care, especially by telephone can exclude people with IDD who may not understand the context, recognize the speaker or be able to easily express themselves (Selick et al. 2022; Hatton et al. 2024). There are also challenges in terms of confidentiality and infrastructure within disability services to participate fully in virtual care and challenges with regard to digital literacy that need to be addressed (Keenan and Doody 2023). It is important for the policy changes that made virtual care more accessible for adults with IDD to persist beyond the pandemic, but that decisions around who gets virtual care and how it gets delivered be based on individual needs, preferences and skills. Both patients and providers need additional training to ensure this type of care is accessible to this group.

For adults with IDD, monitoring the probability of using care and quality of care during nonpandemic times can serve as a baseline to contextualize our understanding of changes to healthcare in future public health emergencies. They are an important group to monitor even after the acute part of the COVID pandemic has passed because they have higher rates of health issues and of healthcare use than others without IDD, so large‐scale changes to health risks or healthcare delivery (both of which occurred the acute part of the COVID pandemic) may affect them more than others. Moreover, the effects of these changes may have a lagged effect, so continued monitoring is important.

### 4.1 Strengths and Limitations

Assessing population‐based groups from the same geopolitical area using multiple, identically defined outcomes with two full years of data from the start of the pandemic is a study strength. A key limitation is that this analysis did not account for age and sex difference in adults with IDD versus no IDD; adults with IDD in Ontario are much younger, more likely to be male than others (Durbin et al. 2022). Also, because administrative healthcare datasets were used, key information was not available on residential setting (e.g., congregate care), quality of care received, the nature of provider–patient interactions, patient preference and need. Finally, these data could not differentiate between phone‐based and video‐based virtual care as that coding distinction was only made in Ontario starting in October 2021.

### 4.2 Summary

Using objective records of healthcare use, this paper reports a reduction in use of most healthcare services during the first COVID‐19 year with increases in year 2, and a dramatic increase in virtual in‐office physician visits for adults with IDD and those without IDD in year 2. A suggestion for future work is to account for age differences across groups as the IDD group was younger than the group with no IDD. Future research could also disaggregate visits to physicians by physician specialty and by type of virtual care as people with IDD may be more likely than others to use phone rather than video services although phone‐based care is less effective for them. Additionally, research combining care quantity with care quality would be important.

## Ethics Statement

The use of the data in this project is authorized under Section 45 of Ontario's Personal Health Information Protection Act (PHIPA) and does not require review by a research ethics board.

## Conflicts of Interest

The authors declare no conflicts of interest.

## Data Availability

The data set from this study is held securely in coded form at ICES. The authors accessed the data set used for this study in a manner that is different from the manner by which individuals who are external to ICES would access the data set. The authors are affiliated with ICES, either directly or as collaborating agents of ICES, and conducted the study in fulfilment of ICES' mandate as a prescribed entity under Ontario's Personal Health Information Protection Act. As a result, the authors were authorized, both legally and contractually, to access the data set in a more granular form than individuals who are external to ICES would be permitted to access the data set. External individuals must apply for access to the minimal data to das@ices.on.ca through ICES' Data and Analytic Services, a division of ICES established specifically to provide data and analytic services to third‐party researchers. The data set that approved third‐party researchers would be permitted to access will be adjusted to ensure the risk of re‐identification of any underlying individuals is low. The analytic code is not necessary to replicate the study results because the specific diagnostic codes and definitions of study groups are listed in the appendix, and other necessary details are provided in the Methods section. However, third‐party researchers who wish to replicate the results may still request the analytic code from the authors.

## Appendix A Details on Health Databases Utilized for This Study

**Table:**

Data type	Database name (name acronym)	Data use
Health care registry (demographic and mortality information)	Registered Person's Database (RPDB)	❶ To identify people eligible with Ontario health card numbers (i.e., had provincial universal health insurance coverage) who were eligible for the study
Physician claims (virtual visits, in office visits)	Ontario Health insurance Plan (OHIP)	❶ To determine membership in the group with or without IDD
		❷ To determine service use
Home care services	Home Care Database (HCD)	❶ To determine service use
Census geographic identifier	Postal Code Conversion File (PCCF)	❶ To classify the level of rurality of the person's residential neighbourhood
Emergency department visits	National Ambulatory Care Reporting System (NACRS)	❶ To determine service use
General hospital admissions	Canadian Institutes of Health Information Discharge Abstract Database (CIHI‐DAD)	
Mental illness and addiction hospital admissions	Ontario Mental Health Reporting System (OMHRS)	

## Appendix B Definition of Developmental Disability Diagnosis

**Table:** **TABLE B.1.** Diagnostic codes used to identify individuals with developmental disabilities in the administrative health data (from Atlas on the Primary Care of Adults with Developmental Disabilities in Ontario).

Database	Year of inception	Diagnoses	Criteria
Discharge Abstract Database	1988	Discharges with any diagnosis listed in Table B.2	• In any diagnostic field • For all facilities submitting to DAD, SDS and NACRS • From inception of database to March 15, 2022
Same Day Surgery Database	1991		
National Ambulatory Care Reporting System	2002		
Ontario Mental Health Reporting System	2005	299 to 299.80, 317 to 319.99	• For all facilities submitting to OMHRS • From inception of database to March 15, 2022
Ontario Health Insurance Plan	1991	299, 319	• For all providers submitting to OHIP • From June 1991 to March 15, 2022

**TABLE B.2:** Developmental disabilities and related codes included in the International Classification of Diseases, 9th and 10th editions.

Code	Label
**ICD‐9**	
299–299.99	Pervasive developmental disorders (e.g., autism)
317–317.99	Mental retardation
318–318.99	Mental retardation
319–319.99	Mental retardation
758.0–758.39	Chromosomal anomalies for which a developmental disability is typically present
758.5	Other conditions due to autosomal anomalies
758.8, 758.89	Other conditions due to chromosome anomalies (do not include 758.81)
758.9	Conditions due to anomaly of unspecified chromosome
759.5	Tuberous sclerosis
759.81	Other and unspecified congenital anomalies: Prader–Willi syndrome
759.821	Other and unspecified congenital anomalies: de Lange syndrome (include only if 6 digits exist; i.e., do not include 759.82)
759.827	Other and unspecified congenital anomalies: Seckel syndrome (include only if 6 digits exist)
759.828	Other and unspecified congenital anomalies: Smith–Lemli–Opitz syndrome (include only if 6 digits exist)
759.83	Other and unspecified congenital anomalies: Fragile X syndrome
759.874	Other and unspecified congenital anomalies: Beckwith–Wiedemann syndrome (include only if 6 digits exist)
759.875	Other and unspecified congenital anomalies: Zellweger syndrome (include only if 6 digits exist)
759.89	Other and unspecified congenital anomalies: other (e.g., Menkes disease, Laurence–Moon–Biedl syndrome, Rubinstein–Taybi syndrome)
760.71	Fetal alcohol syndrome
760.77	Fetal hydantoin syndrome
**ICD‐10**	
F700	Mild mental retardation with the statement of no, or minimal, impairment of behaviour
F701	Mild mental retardation, significant impairment of behaviour requiring attention or treatment
F708	Mild mental retardation, other impairments of behaviour
F709	Mild mental retardation without mention of impairment of behaviour
F710	Moderate mental retardation with the statement of no, or minimal, impairment of behaviour
F711	Moderate mental retardation, significant impairment of behaviour requiring attention or treatment
F718	Moderate mental retardation, other impairments of behaviour
F719	Moderate mental retardation without mention of impairment of behaviour
F720	Severe mental retardation with the statement of no, or minimal, impairment of behaviour
F721	Severe mental retardation, significant impairment of behaviour requiring attention or treatment
F728	Severe mental retardation, other impairments of behaviour
F729	Severe mental retardation without mention of impairment of behaviour
F730	Profound mental retardation with the statement of no, or minimal, impairment of behaviour
F731	Profound mental retardation, significant impairment of behaviour requiring attention or treatment
F738	Profound mental retardation, other impairments of behaviour
F739	Profound mental retardation without mention of impairment of behaviour
F780	Other mental retardation with the statement of no, or minimal, impairment of behaviour
F781	Other mental retardation, significant impairment of behaviour requiring attention or treatment
F788	Other mental retardation, other impairments of behaviour
F789	Other mental retardation without mention of impairment of behaviour
F790	Unspecified mental retardation with the statement of no, or minimal, impairment of behaviour
F791	Unspecified mental retardation, significant impairment of behaviour requiring attention or treatment
F798	Unspecified mental retardation, other impairments of behaviour
F799	Unspecified mental retardation without mention of impairment of behaviour
F840	Childhood autism
F841	Atypical autism
F843	Other childhood disintegrative disorder
F844	Overactive disorder associated with mental retardation and stereotyped movements
F845	Asperger's syndrome
F848	Other pervasive developmental disorders
F849	Pervasive developmental disorder, unspecified
Q851	Tuberous sclerosis
Q860	Fetal alcohol syndrome
Q861	Fetal hydantoin syndrome
Q871	Aarskog, Prader–Willi, deLange, Seckel, etc.
Q8723	Rubinstein–Taybi syndrome (include only if all 5 digits)
Q8731	Sotos syndrome (include only if all 5 digits)
Q878	Other
Q900–Q939 except Q926	All Down syndrome types, cri du chat, etc., except extra marker chromosomes
Q971	Female with more than three X chromosomes
Q992	Fragile X syndrome
Q998	Other specified chromosome abnormalities
